# Supplementary material for: Multiheme selenoenzyme essential for elemental sulfur respiration
Source: Sci Adv. 2026 Aug 26;12(35):eaeg2218. doi: 10.1126/sciadv.aeg2218 (PMC13510729; doi:10.1126/sciadv.aeg2218)
Supplement: Supplementary file 1 [file sciadv.aeg2218_sm.pdf]

Science Advances
AAAS

Supplementary Materials for

Multiheme selenoenzyme essential for elemental sulfur respiration

Hisaaki Mihara *et al.*

Corresponding author: Hisaaki Mihara, mihara@fc.ritsumei.ac.jp;
Hiroyoshi Matsumura, h-matsu@fc.ritsumei.ac.jp

Sci. Adv. **12**, eaeg2218 (2026)
DOI: 10.1126/sciadv.aeg2218

The PDF file includes:

Figs. S1 to S8
Tables S1 to S3
Legends for data S1 to S5

Other Supplementary Material for this manuscript includes the following:

Data S1 to S5

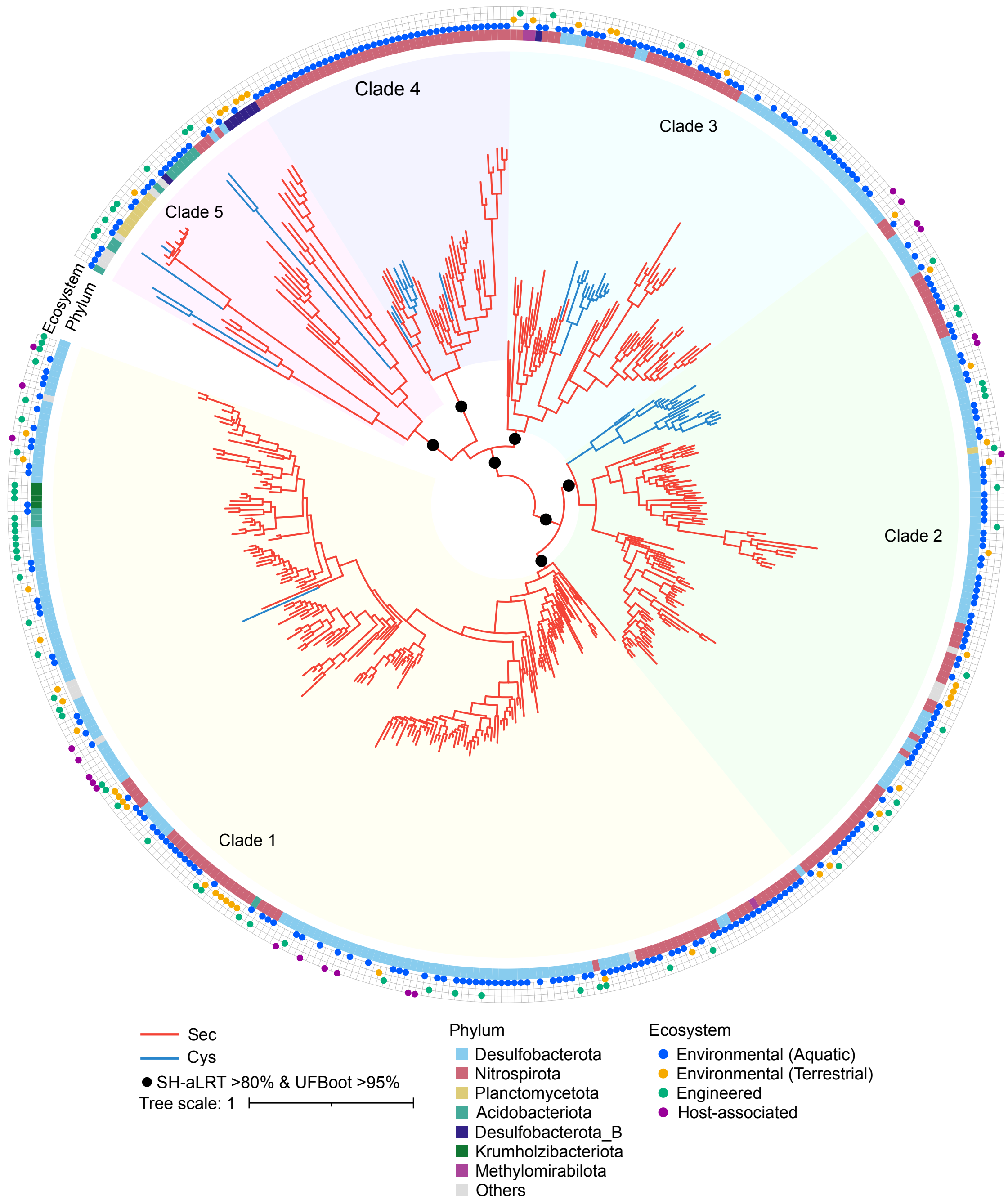

Fig. S1. Unrooted maximum-likelihood phylogenetic tree of the MccSep family with associated taxonomy and ecosystem information related to Fig. 1.

The tree was constructed from a multiple sequence alignment of centroid sequences representing 455 clusters at 90% amino acid sequence identity. Branches containing Sec- and Cys-type MccSep homologs are colored red and blue, respectively. Major clades (1–5) are shaded in different colors. Black circles indicate branches separating major clades with bootstrap values of SH-aLRT >0.80 and UFBoot >0.95. Colored strips in the inner ring represent phylum-level taxonomy. Colored circles in the four outer rings represent ecosystem categories.

```
   1 ATGAAGCACAAAACCGCATGGCTGACCCTTGCTGCCGCAGCGCTTGCGCTCTGTGCCGCCGCACCGGTTTTTGCCGAAAAGGCGGGCATCGGCTGGCAGGAGACG
   1  M  K  H  K  T  A  W  L  T  L  A  A  A  A  L  A  L  C  A  A  A  P  V  F  A  E  K  A  G  I  G  W  Q  E  T

 106 ATCGTTGCCAAGAGCGGCAAGGCAAAGACCATGGCCGAGTTGGCAAAGATGTATGATTCCAGCTCCTGCATCGAGTGTCACCAGGAAGTCCACGATGAATGGGAG
  36  I  V  A  K  S  G  K  A  K  T  M  A  E  L  A  K  M  Y  D  S  S  S  C  I  E  C  H  Q  E  V  H  D  E  W  E
                                                                      Heme 1

 211 CAGTCGATCCATGCCCGCTCAATCTTCGGTACCGGCCGGACTGCCGCCACCTTCATGACTGCTGTGGTCAACGGCCTCATGGAATGGGAATATTCGGGGGTGAAA
  71  Q  S  I  H  A  R  S  I  F  G  T  G  R  T  A  A  T  F  M  T  A  V  V  N  G  L  M  E  W  E  Y  S  G  V  K

 316 AGCCCGTCGGACGTGAAAGTTGAGCACCTGATGGGATGCGCCAAGTGCCATCTCCCCCAACTGGCCGACGCCGAGGACTCGGTGGCCAAGGAGATCATCTCCACC
 106  S  P  S  D  V  K  V  E  H  L  M  G  C  A  K  C  H  L  P  Q  L  A  D  A  E  D  S  V  A  K  E  I  I  S  T
                                          Heme 2

 421 ATCGGCAGCTGGCAGGATGCACTGAGGAAAAAGGATTCGGCAAAGGCGGTTGAAGAGGCCGACAAGCTCAAGAGCCTCAACATCAATTGCCTCGTTTGCCATAAC
 141  I  G  S  W  Q  D  A  L  R  K  K  D  S  A  K  A  V  E  E  A  D  K  L  K  S  L  N  I  N  C  L  V  C  H  N
                                                                                             Heme 3

 526 CGTAACGCCATCACCCACAAGTGGACCGACGGCTATCCCCAGGCCGGGGTAGTCTACGGCTCCAAGGAAGGGGAACACCCCTCTGCGGCATTCCCCACGATGAAG
 176  R  N  A  I  T  H  K  W  T  D  G  Y  P  Q  A  G  V  V  Y  G  S  K  E  G  E  H  P  S  A  A  F  P  T  M  K

 631 GTCAGTCCGATCATGTCAGAGTCCATCCAGTGCGGCCAATGCCACGGCTTGGGGCCGAACCTGGAATTGGATAACCCGACCCAGTGCTGCACCAGCTATGCGAGC
 211  V  S  P  I  M  S  E  S  I  Q  C  G  Q  C  H  G  L  G  P  N  L  E  L  D  N  P  T  Q  C  C  T  S  Y  A  S
                                    Heme 4

 736 TACCTCTGGGCCTACAAGGCCGAGGGCGGGCGGGAAAACTGCCAGGAATGCCACATGAAGAAGAGCAAGCTCGGGCACAACATGCAGAGCTACCGTGACCCGGGC
 246  Y  L  W  A  Y  K  A  E  G  G  R  E  N  C  Q  E  C  H  M  K  K  S  K  L  G  H  N  M  Q  S  Y  R  D  P  G
                                             Heme 5

 841 ATGGCCAAGGCCGCCGTTGAGTTCAAGGCCGAGGCCTATGGCTATCACTGGCGGGACGGGGCGCTGGTGACGCCCAAGGCAGTGGTGAAGGTCGAGATGACCAAT
 281  M  A  K  A  A  V  E  F  K  A  E  A  Y  G  Y  H  W  R  D  G  A  L  V  T  P  K  A  V  V  K  V  E  M  T  N

 946 CACGCGGGCCACTCGATCCCCGATGGCTGACCGACCCCCAACCGACTGGTTCTGTCGGTCATTGCAAAGACAAAAGACGGCGAAGAAGTGTTCAATCAGGAAAAG
 316  H  A  G  H  S  I  P  D  G  U  P  T  P  N  R  L  V  L  S  V  I  A  K  T  K  D  G  E  E  V  F  N  Q  E  K

1051 ATCTACATGCCGGTGCCCCAGCAGCTGGCCCGGGGTGACCGGATGGGGCGCGGCCCCTATGAGAAAAGCGGTATGATCGAGGATACGGGTCTGCCGCCGGGCAAG
 351  I  Y  M  P  V  P  Q  Q  L  A  R  G  D  R  M  G  R  G  P  Y  E  K  S  G  M  I  E  D  T  G  L  P  P  G  K

1156 AAGATCCACGAGCGCTTCGATATCCTCTTCCCCACCGAGGATGTGGTGGAGGACGGGAAGAAGGTTCGCAAGACCCTGGCCCACGATCTGGAAGTGGAGGTGAAA
 386  K  I  H  E  R  F  D  I  L  F  P  T  E  D  V  V  E  D  G  K  K  V  R  K  T  L  A  H  D  L  E  V  E  V  K

1261 CTCTGGTATCTCCCCTTCGGTTCAATGAATTCCGATCCGTTCCTCTGGCATGAGTTCACCCAGAAGGTGAGCATCAGCGCCAAGGGCAAGTAG
 421  L  W  Y  L  P  F  G  S  M  N  S  D  P  F  L  W  H  E  F  T  Q  K  V  S  I  S  A  K  G  K  *
```

Fig. S2. Nucleotide and amino acid sequences of MccSep.

The 81-nt region flanking *gsu2937* and *gsu2936* is underlined. The TGA codon encoding selenocysteine (U) at the amino acid position 325 is shown in blue. The erroneously annotated start codon of *gsu2936* and the corresponding methionine residue are indicated by magenta. The postulated SECIS element is indicated by underlined red letters. The N-terminal signal peptide (1–25) is shaded in gray. Five heme-binding motifs (CxxCH) and heme numbering are highlighted in cyan. The nucleotide and amino acid sequences of MccSep were retrieved from the NCBI database (accession numbers: GS_RS14775 and WP_262421250.1, respectively).

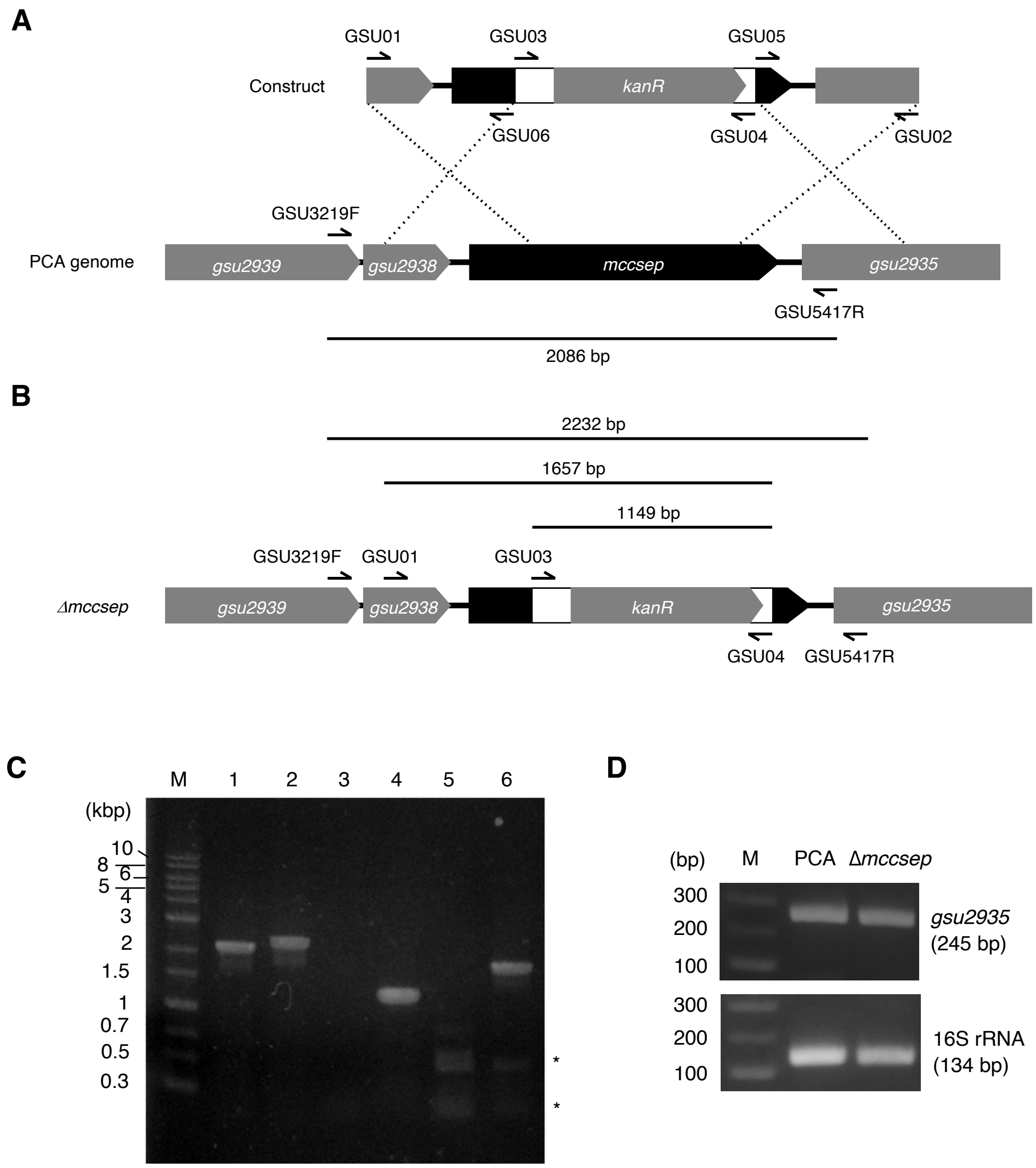

Fig. S3. Construction of an *mccsep*-deficient strain (Δ*mccsep*).

(**A**) Schematic drawing of the homologous recombination between the disruption construct and the genomic DNA of the parent strain *G. sulfurreducens* PCA (PCA). Primer annealing sites are indicated. (**B**) Schematic drawing of the corresponding genomic region of Δ*mccsep*. Primer annealing sites and predicted sizes of PCR fragments are indicated. (**C**) PCR analysis using genomic DNAs from the parent PCA strain (lanes 1, 3, and 5) and Δ*mccsep* (lanes 2, 4, and 6). Primers used were GSU3219F and GSU5417R (lanes 1 and 2), GSU03 and GSU04 (lanes 3 and 4), and GSU01 and GSU04 (lanes 5 and 6). Asterisks indicate non-specific bands. (**D**) Reverse transcription-PCR analysis showing no negative polar effect on the downstream gene expression by the *mccsep* disruption. mRNA levels of *gsu2935* and 16S rRNA in the parent PCA and Δ*mccsep* strains were analyzed by RT-PCR using each primer set (gsu2935-F/gsu2935-R and 16SrRNA-F/16SrRNA-R, respectively) shown in table S3. Cells were grown in NBAFYE medium at 35 ºC to the mid-exponential phase.

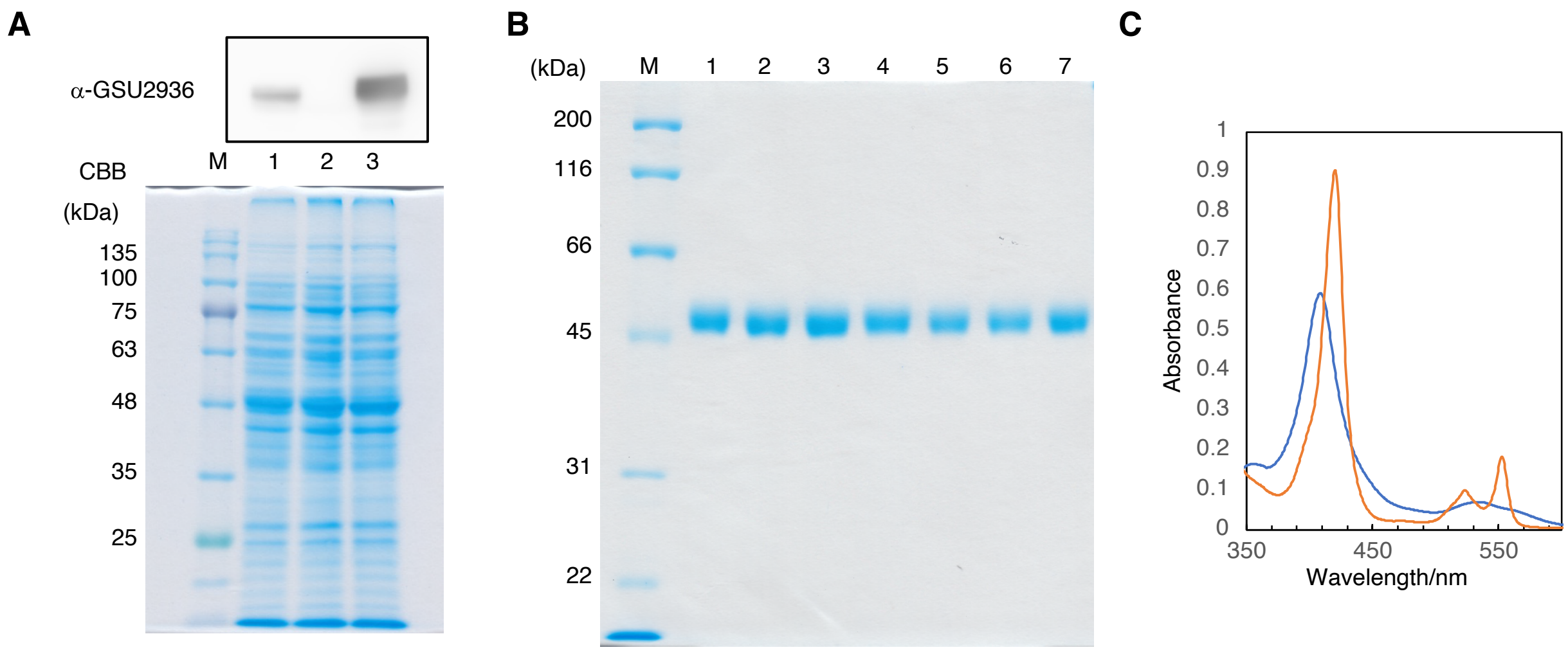

Fig. S4. Expression and purification of recombinant MccSep.

(**A**) Western blot analysis of the crude extracts from the parent strain *G. sulfurreducens* PCA (lane 1), Δ*mccsep* (lane 2), and Δ*mccsep* complemented with pMcasMccSepStag carrying the *mccsep* gene (lane 3). Crude extracts of *G. sulfurreducens* strains grown in NBAYFE medium were separated on 10% SDS-PAGE gels and analyzed by western blotting using anti-GSU2936 antibody (upper panel). The lower panel represents the identical gels visualized by CBB staining to show the amount of proteins loaded. (**B**) Purification of recombinant MccSep (lane 1) and its mutants (lanes 2, U325A; 3, U325C; 4, C239H; 5, C240A; 6, R83A; and 7, K372A). Each recombinant enzyme was purified as a Strep-tag fusion protein and analyzed by SDS-PAGE. Proteins were visualized by CBB staining. (**C**) UV–vis absorption spectra of air-oxidized (blue line) and 0.02 mM sodium dithionite-reduced (orange line) MccSep (0.06 mg/mL).

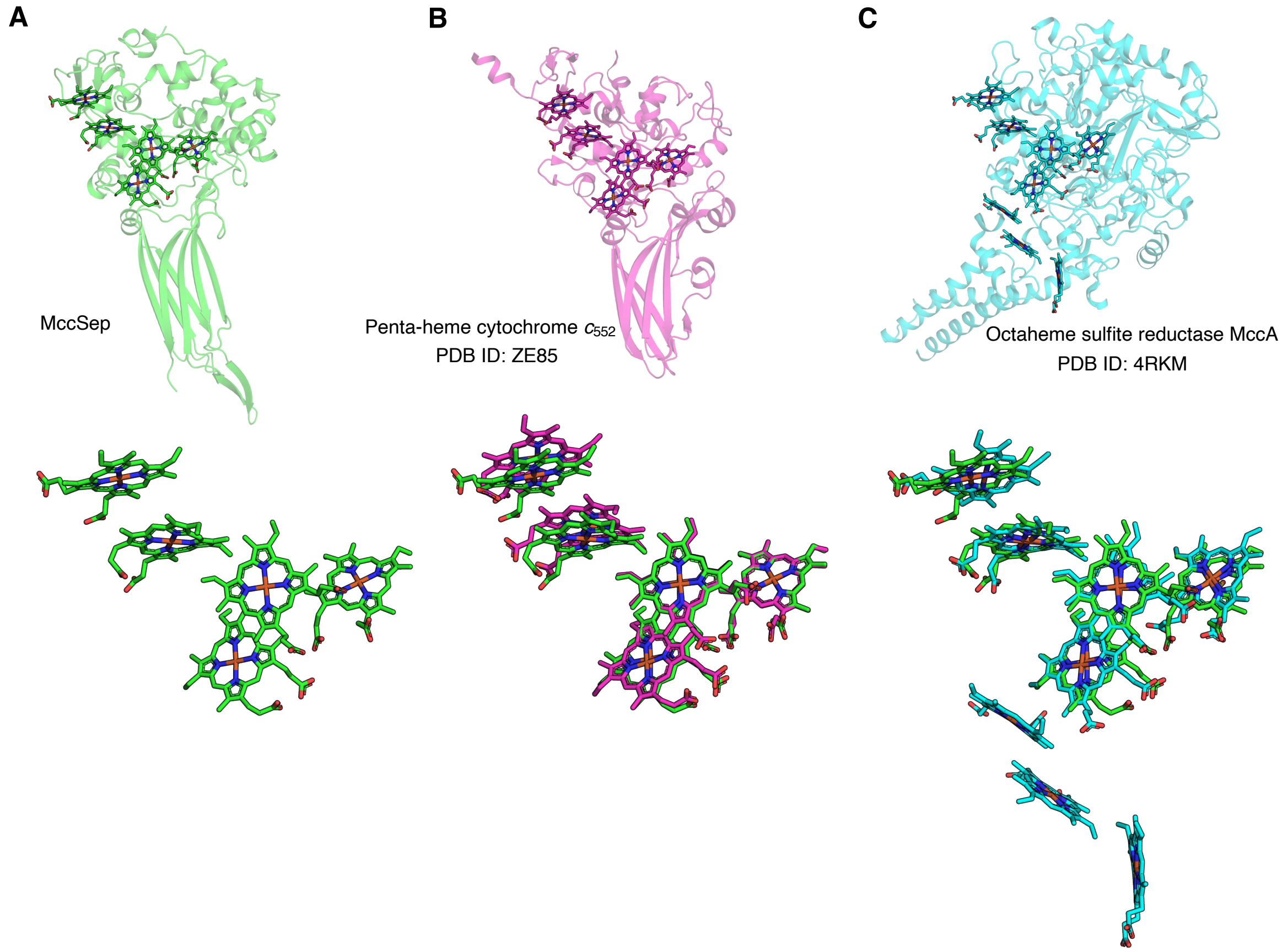

Fig. S5. Comparison with multiheme-binding proteins.

Structural comparison of MccSep with representative multiheme-binding cytochromes. Upper panels show monomeric structures; lower panels display superimposed heme groups aligned with those of MccSep. (**A**) MccSep. (**B**) Penta-heme cytochrome c_{552} from *Thermochromatium tepidum* (PDB ID: 5ZE8). (**C**) Octaheme sulfite reductase MccA from *Wolinella succinogenes* (PDB ID: 4RKM).

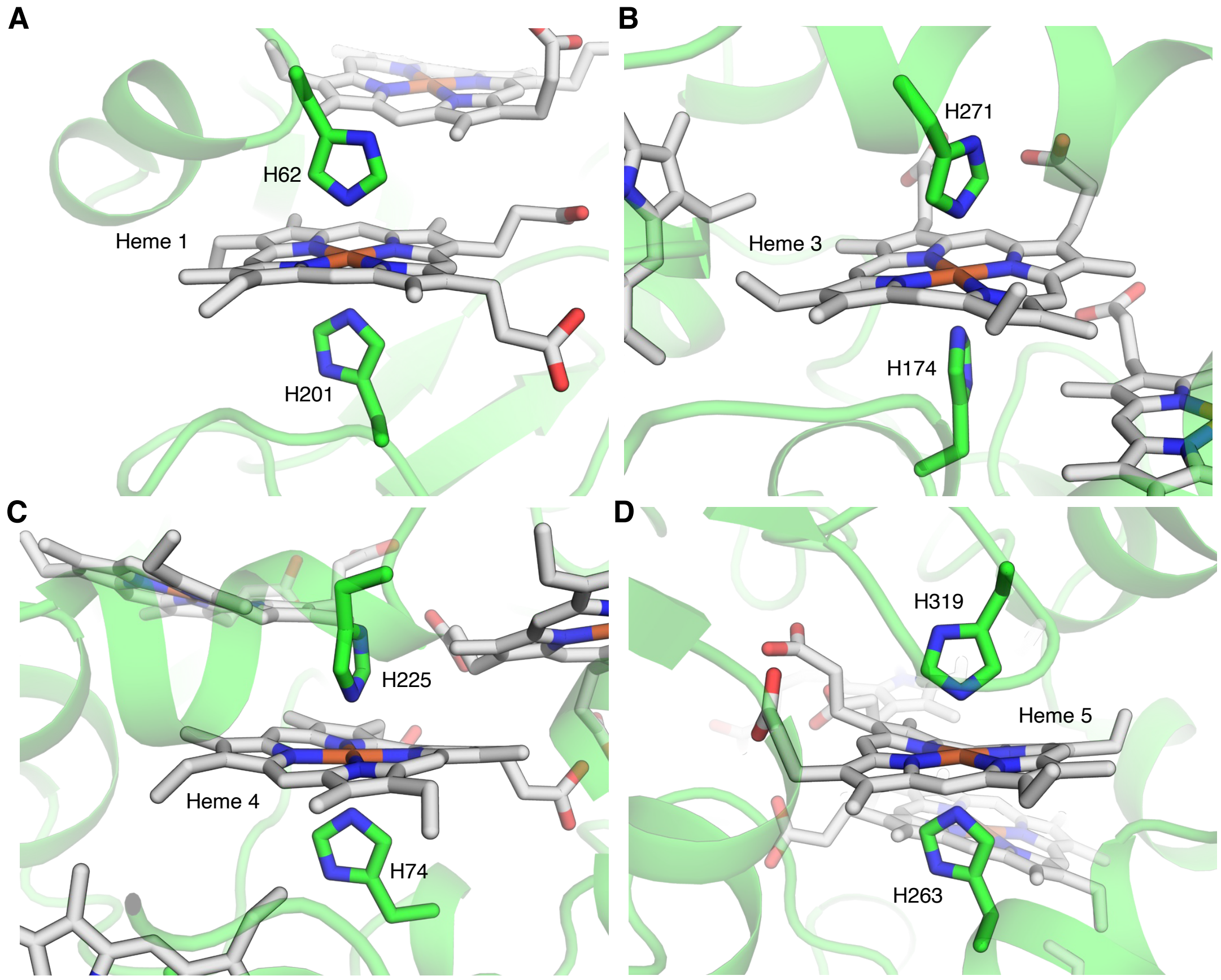

Fig. S6. Heme binding sites in MccSep.

Hemes 1 (**A**), 3 (**B**), 4 (**C**), and 5 (**D**) are coordinated by two histidine residues, forming a bis-histidine axial ligation.

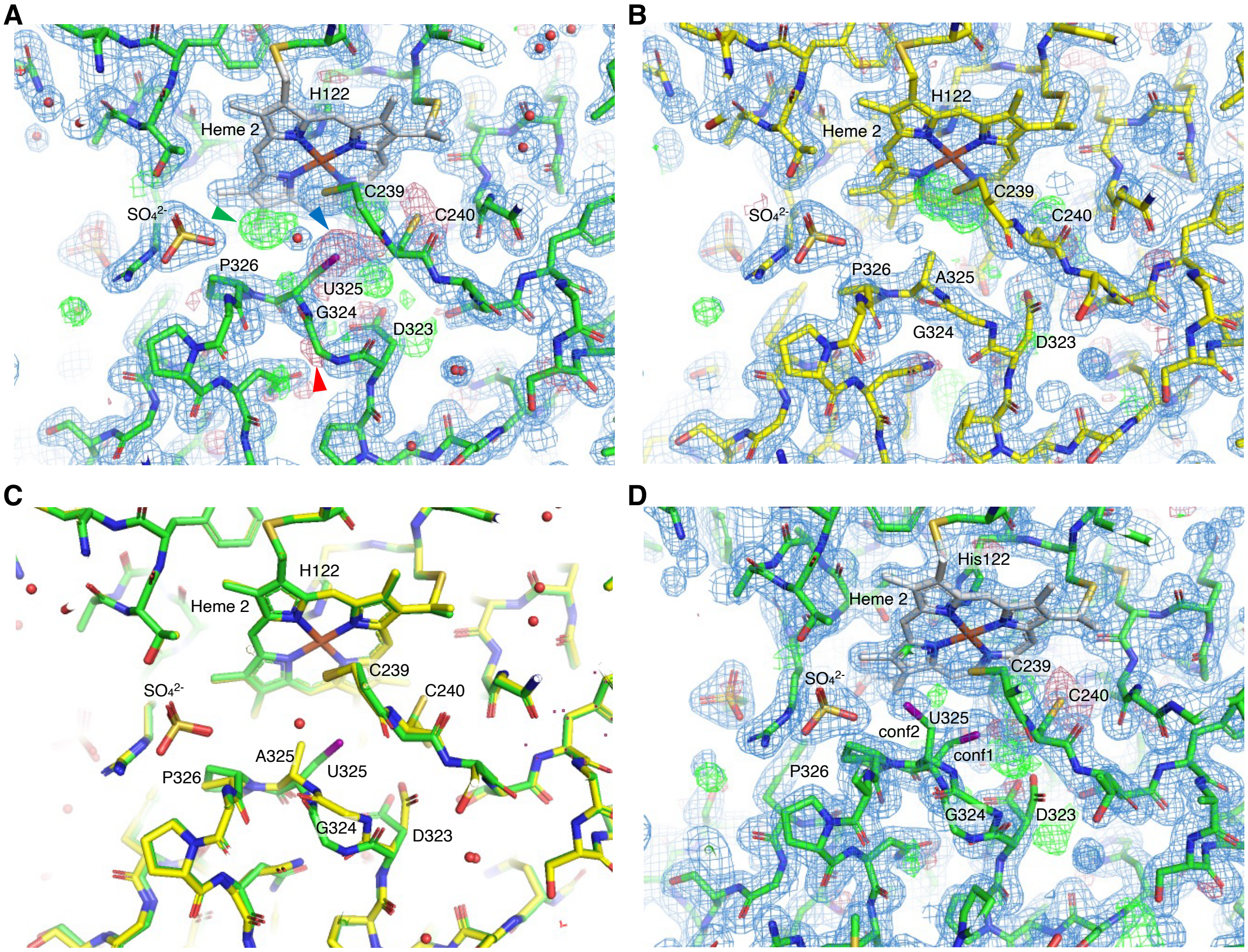

Fig. S7. Assignment of alternative conformations of the 323–326 loop.

(**A**) Initial refinement of the WT MccSep structure showing ambiguous electron density near U325. Negative $F_o - F_c$ electron density peaks are shown on the main chain of G324 (red arrowhead) and the side chain of U325 (blue) with a positive peak ~3.0 Å from the oxygen atom of a sulfate ion (green). (**B**) Initial refinement of the U325A mutant showing that the loop adopts a single conformation with clear electron density, except for a residual positive peak near C239. (**C**) Superposition of the WT and U325A structures revealing differences in the C_α–C_β orientation of residue 325, the main chain of G324, and the carbonyl group of D323. (**D**) Partially overlapping $2F_o - F_c$ electron densities in the WT MccSep structure with those in the U325A structure suggest alternative loop conformations in WT. Refinement of the WT structure with dual conformations improved map interpretability. All maps: $2F_o - F_c$ (blue, 1.2σ); $F_o - F_c$ positive (green, 4σ); $F_o - F_c$ negative (red, –3σ).

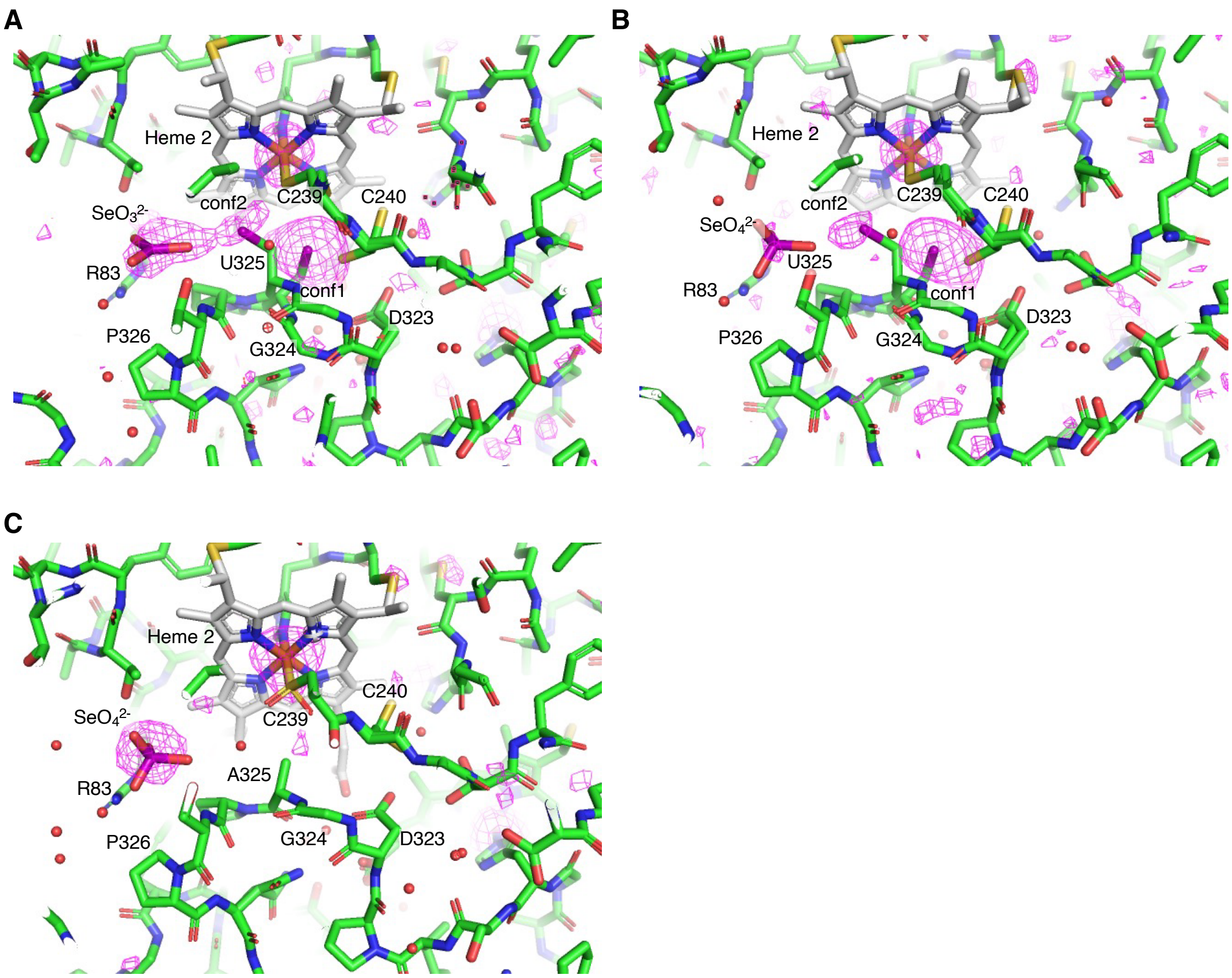

Fig. S8. Anomalous difference Fourier maps at the active site.

(**A–C**) Anomalous difference Fourier maps (magenta, 2.5σ, λ = 0.9792 Å) for WT MccSep soaked with 10 mM selenite overnight (**A**), 10 mM selenate overnight (**B**), and for U325A soaked with 100 mM selenate (**C**). Anomalous difference Fourier map for WT MccSep soaked with 10 mM selenite (**A**) shows a continuous electron density between U325 in conf2 and bound selenite.

Table S1. Apparent kinetic parameters for polysulfide-dependent MV oxidation by MccSep and its mutants

Data are shown as means ± standard errors of triplicate measurements.

[a]Hill coefficient

[b]n.d.: not determined due to no detectable activity

Enzyme	Apparent V_{max} (μmol $MV^{•+}$ min^{-1} mg^{-1})	Apparent $K_{0.5}$ (mM)	h^{a}	Apparent $V_{max}/K_{0.5}$ (μmol $MV^{•+}$ min^{-1} mg^{-1} mM^{-1})
MccSep	83 ± 7.0	0.19 ± 0.023	1.9 ± 0.31	(4.4 ± 0.66) × 10^2
R83A	16 ± 0.78	0.21 ± 0.011	3.8 ± 0.69	73 ± 5.3
C239H	n.d.[b]	n.d.[b]	n.d.[b]	n.d.[b]
C240A	70 ± 4.8	0.14 ± 0.015	2.1 ± 0.45	(4.9 ± 0.61) × 10^2
U325A	n.d.[b]	n.d.[b]	n.d.[b]	n.d.[b]
U325C	n.d.[b]	n.d.[b]	n.d.[b]	n.d.[b]
K372A	5.6 ± 0.27	0.074 ± 0.0088	2.0 ± 0.48	76 ± 9.7

Table S2. Data collection, phasing, and refinement statistics

*Each dataset was collected from one crystal. Values in parentheses are for the highest-resolution shell.

	WT native	WT I-derivative	U325A	WT-selenite	WT-selenate	U325A-selenate
PDB accession code	9WT8		9WT9	9WTA	9WTB	9WTC
**Data collection**						
Space group	$P2_1$	$P2_1$	$P2_1$	$P2_1$	$P2_1$	$P2_1$
Cell dimensions						
a, *b*, *c* (Å)	83.03 123.54 116.52	83.34, 122.98, 116.76	83.17 122.65 116.45	83.45 123.56 116.61	83.64 123.83 116.62	83.39 123.62 116.64
β (°)	92.19	92.28	92.15	92.15	91.94	91.60
Resolution (Å)	48.54 - 1.75 (1.81 - 1.75) *	50.00 - 2.40 (2.46 - 2.40) *	48.53 - 1.80 (1.86 - 1.80) *	45.25 - 2.20 (2.28 - 2.20) *	46.10 - 2.20 (2.28 - 2.20) *	42.41 - 2.20 (2.28 - 2.20) *
R_{sym} or R_{merge}	0.063 (0.981)	0.061 (0.981)	0.081 (1.931)	0.081 (0.934)	0.083 (0.810)	0.072 (0.899)
$I / \sigma I$	18.12 (2.03)	21.75 (6.85)	14.33 (0.90)	17.58 (2.20)	16.45 (2.38)	19.89 (2.32)
Completeness (%)	99.3(98.6)	95.1 (91.8)	99.8 (99.7)	99.9 (100.0)	99.9 (100.0)	99.9 (100.0)
Redundancy	7.1 (7.4)	14.6 (15.0)	7.0 (7.3)	6.9 (7.1)	6.9 (7.1)	6.9 (7.1)
**Refinement**						
Resolution (Å)	48.54 - 1.75 (1.81 - 1.75) *		48.53 - 1.80 (1.86 - 1.80) *	45.25 - 2.20 (2.28 - 2.20) *	46.10 - 2.20 (2.28 - 2.20) *	42.41 - 2.20 (2.28 - 2.20) *
No. reflections	234,125 (23,146)		215,139 (21,395)	119,604 (11,857)	120,174 (11,920)	119,673 (11,904)
R_{work} / R_{free}	0.1669 (0.2865) / 0.1981 (0.3149)		0.1870 (0.3828) / 0.2243 (0.4084)	0.1646 (0.2823) / 0.2066 (0.3186)	0.1644 (0.2654) / 0.2104 (0.3000)	0.1682 (0.2846) / 0.2140 (0.3255)
No. atoms						
Protein	13,426		13,218	13,429	13,445	13,265
Ligand/ion	1,458		1,181	1,224	1,195	1,063
Water	1,141		1,038	962	949	916
B-factors						
Protein	33.27		39.89	46.38	44.82	46.11
Ligand/ion	34.98		39.78	46.51	44.26	43.98
Water	41.85		44.87	49.99	48.46	46.36
R.m.s deviations						
Bond lengths (Å)	0.009		0.009	0.009	0.009	0.009
Bond angles (°)	1.05		1.06	0.96	0.97	0.98

Table S3. Oligonucleotides used

Primer	Sequence (5´ to 3´)
gsu2935-F	AATGCTACGGCTGTCATACGAAATA
gsu2935-R	TTTCCCCTTGAAGGTAGAGACGTAG
16SrRNA-F	TGAGACACGGTCCAGACTCCTAC
16SrRNA-R	TCA TTTCTTCCCTCCCGACA
GSU01	AGAAGGGCGCCCATGTTC
GSU02	TGATCCCCGTATTGGTAT
GSU03	TCGATCCATGCCCGCTCAAGGATGAATGTCAGCTAC
GSU04	TCCACTTCCAGATCGTGGAGAAGGCGGCGGTGGAATCG
GSU05	CCACGATCTGGAAGTGGA
GSU06	TGAGCGGGCATGGATCGA
GSU3219F	GGAAGGCACGTACAACGGAGTCAAGAGC
GSU5417R	GCACCCGGCAGCACTAAGGAACATAACG
GSU2936-F	GGAATTCCATATGCCGACCCCCAACCGACTGGTTCTG
GSU2936-R	CTCCGCTCGAGCTTGCCCTTGGCGCTGATG
casF	AATTGACTGACTGAGAAGAAGATATACATATGCCCTCGAGTCTAGAG
casR	AATTCTCTAGACTCGAGGGCATATGTATATCTCCTTCTCAGTCAGTC
MccSep-F	ATGGATCCCATATGAAGCACAAAACCGCATG
MccSep-Stag-R	TTTTTCGAACTGCGGGTGGCTCCACCTACCTTCGATCTTGCCCTTGGCGCT
Stag-Xba-R	AATTTCTAGATTATTTTTCGAACTGCGGGTGGCTCCA
MccSep-R	AATTTCTAGACTACTTGCCCTTGGCGCTGATGC
C325A-F	ATCCCCGATGGCTGCCCGACCCCCAACCG
C325A-R	CGGTTGGGGGTCGGGCAGCCATCGGGGAT
C325C-F	CGATCCCCGATGGCGCCCCGACCCCCAACC
C325C-R	GGTTGGGGGTCGGGGCGCCATCGGGGATCG
C240A-F	CCGACCCAGTGCGCCACCAGCTATGCGAGC
C240A-R	GCTCGCATAGCTGGTGGCGCACTGGGTCGG

List of supplementary excel files:

Data S1. (separate file)

Genomic, protein, and ecosystem information of the 475 Sec-type MccSep homologs obtained from the UGA stop-codon read-through analysis in this study. There are 394 non-redundant protein sequences.

Data S2. (separate file)

Genomic, protein, and ecosystem information of the 208 Sec- and 89 Cys-type MccSep homologs obtained from the NCBI database. There are 175 Sec- and 68 Cys-type non-redundant protein sequences.

Data S3. (separate file)

Activity of WT MccSep with various compounds.

Data S4. (separate file)

Assay of C239H, U325A, and U325C with polysulfide.

Data S5. (separate file)

Assay of R83A, C239H, K372A, U325A, and U325C with selenite.
